# Case Report: De novo variant in myelin regulatory factor in a Chinese child with 46,XY disorder/difference of sex development, cardiac and urogenital anomalies, and short stature

**DOI:** 10.3389/fped.2022.1027832

**Published:** 2022-11-18

**Authors:** Hui Wang, Dian Wu, De-Hua Wu, Hong-Juan Tian, Hai-Feng Li, Ke-Wen Jiang, Chao-Chun Zou

**Affiliations:** ^1^ Department of Rehabilitation, The Children’s Hospital, Zhejiang University School of Medicine, National Clinical Research Center for Child Health, Hangzhou, China; ^2^ Department of Psychological, The Children’s Hospital, Zhejiang University School of Medicine, National Clinical Research Center for Child Health, Hangzhou, China; ^3^ Department of Urology, The Children’s Hospital, Zhejiang University School of Medicine, National Clinical Research Center for Child Health, Hangzhou, China; ^4^ Department of Endocrinology, The Children’s Hospital, Zhejiang University School of Medicine, National Clinical Research Center for Child Health, Hangzhou, China

**Keywords:** *MYRF* gene, cardiac-urogenital syndrome, disorders/differences of sex development, nanophthalmos, short stature

## Abstract

The myelin regulatory factor (*MYRF*; MIM# 608329) gene was first identified as a critical transcription factor involved in oligodendrocyte differentiation and central nervous system myelination. With the recent development of exome sequencing, pathogenic variants of *MYRF* had been considered as the cause of cardiac-urogenital syndrome (CUGS), 46,XY and 46,XX disorders/differences of sex development (DSDs), and nanophthalmos. Herein, we described a 4-year-7-month-old “girl” with ventricular septal defect, atrial septal defect, patent ductus arteriosus, severe pulmonary hypertension, moderate-to-severe tricuspid regurgitation, enlarged coronary sinus, left superior vena cava, and right lung hypoplasia at birth. Later, the patient developed short stature and amblyopia. Further examination revealed a karyotype 46,XY and visible uterus, whereas the presence of gonads were not explored. Laparoscopy revealed dysplasia of testicular gonad. Whole-exome sequencing (WES) was performed and a *de novo* heterozygous mutation in *MYRF* was identified, known as c.2817G > A/p. W939* (NM_001127392.3). Therefore, this case report presented multiple clinical manifestations with syndromic symptoms of CUGS, 46,XY DSD, and ocular symptoms. These new data expanded the phenotype of the *MYRF* variant and may benefit to characterize the phenotypes caused by the variants of this gene.

## Introduction

Myelin regulatory factor (*MYRF*)-related disorders, including cardiac-urogenital syndrome (CUGS, MIM#618280) and wild encephalitis/encephalopathy with reversible myelin vacuolization (MMERV, MIM#618113), are caused by heterozygous mutations in the *MYRF* (MIM#608329) gene on chromosome 11q12. The *MYRF* gene was first identified as an essential transcription factor for oligodendrocyte differentiation and central nervous system myelination (1–4). Kurahashi et al. (5) identified a heterozygous c.1208A > G transition (NM_001127392) in the *MYRF* gene, resulting in a p.Q403R substitution at a highly conserved residue in the DNA-binding domain, in 9 individuals from 2 different families who had mild encephalitis/encephalopathy and reversible myelin vacuolization in Japan.

In addition to the central nervous system, *MYRF* is also expressed in the heart, lungs, diaphragm, and genitourinary tract. A series of *de novo* heterozygous mutations in *MYRF* were identified in patients with CUGS (6–10). The symptoms of CUGS included partial anomalous pulmonary venous return associated with tracheal anomalies, pulmonary hypoplasia, congenital diaphragmatic hernia (CDH), thyroid fibrosis, thymic involution, cleft spleen, penoscrotal hypospadias, and cryptorchidism (6). A few patients also suffered from short stature, speech delay, intellectual disability and motor delay, and intestinal malrotation. Hamanaka et al. (11) found *MYRF* playing a key role in gonadal cells and tissues development such as Müllerian derivatives, ovaries, Sertoli cells, and Leydig cells. They demonstrated that *MYRF* haploinsufficiency caused a type of disorders/differences of sex development (DSDs) (11). In addition, other studies established *MYRF* as a nanophthalmos gene (12) and the correlation between *MYRF* truncation mutation and high hyperopia (13). These reports proved that *MYRF* played a role in the development of many other organs in addition to regulating cardiac and urogenital development.

Herein, we first reported the case of a male patient with a deleterious *MYRF* variant causing a loss of *MYRF* activity.

## Case presentation

### Case description

A 4-year-7-month-old “girl” was referred to our unit in December 2021 because of the short stature. The current height was 2 standard deviations behind peers; and her height was more than one standard deviation below average in each physical examination. Both eyes were amblyopic. She was her family's second living child (G4P2). The “girl” was born at 36^+5^ weeks, with a birth weight of 2,430 g (*z*-score: −1.91), length of 49 cm (*z*-score: −0.08), and a head circumference of 34.2 cm (*z*-score: 0.27). She underwent a premature cesarean section because of maternal uterine scarring, gestational diabetes, and fetal heart malformation. The Apgar scores were 9 at 1 and 5 min, 5 at 7 min, and 8 at 10 min. After birth, she presented breathing difficulty due to ventricular septal defect (VSD), atrial septal defect (ASD), patent ductus arteriosus (PDA), persistent superior vena cava, and pulmonary hypertension. Besides, the patient also suffered from right-sided pulmonary hypoplasia. She was sent to NICU immediately for respiratory support and other rescue treatments. After 1 month, she underwent surgery to repair ASD, VSD and PDA. She recovered well postoperatively. As the “girl” grows up, the motor and language development was delayed compared to other children of the same age, raising head, climbing, walking and speaking at 6 months, 8 months, 16 months and 18 months respectively. So far, she had been living as a girl since birth.

In her family, 52-year-old father and 17-year-old brother are medium build, her 47-year-old mother has a height of 147 cm and a weight of 48 kg. In addition, her mother had two early pregnancy loss, one due to fetal heart rate disappearance and the other no specific reasons.

Our physical examination showed a weight of 13.5 kg (−1.86 SD), height of 97 cm (−2.21 SD), well-proportioned figure. There was a surgical scar approximately 8 cm long in the median line of the chest. Cardiopulmonary auscultation, nervous system was unremarkable. Both breasts were not enlarged (stage B1). The abdomen was flat and soft, the liver and spleen were not touched under the ribs. Genitourinary physical examination showed female external genitalia with a slightly enlarged clitoris, urethral opening, and vaginal opening in the perineum. No gonadoid masses were found in the inguinal region. No pubic hair was noted. No coffee spot was found all over the body.

The serum levels of sex hormones showed basal luteinizing hormone (LH) of 0.85 IU/L (normal range: <0.32 IU/L), follicle-stimulating hormone (FSH) of 33.15 IU/L (normal range: 0.25–5.89 IU/L), prolactin (PRL) of 242.5 mIU/L (normal range: 108.8–557.0 mIU/L), estradiol (E2) of <36.7 pmol/L (normal range: <115.6 pmol/L), testosterone (T) of 0.46 nmol/L (normal range: <1.31 nmol/L), and human chorionic gonadotropin (HCG) of <1.2 mIU/ml (normal range: <5.0 mIU/ml). Adrenocorticotropic hormone (ACTH) and cortisol at 8 AM were <5 pg/ml (normal range: 0–46 pg/ml) and 92.4 μg/L (normal range: 50–250 μg/L). The blood fasting glucose and insulin level was 4.7 mmol/L (normal range: 3.6–6.11 mmol/L) and 7.5 pmol/L (normal range: 13.0–161.0 pmol/L), respectively. The peak growth hormone (GH) was 14.2 ng/ml (normal range: >10.0 ng/ml) with an insulin-like growth factor 1 (IGF-1) of 41.5 ng/ml (normal range: 35.0–232.0 ng/ml). The SRY gene (14) was positive in peripheral blood examination. Renal function, liver function, electrolytes, thyroid function, alpha fetal protein, carcinoembryonic antigen, and genetic metabolic disease maps were all in the normal range. Interpreting these results in an integrated manner, growth hormone therapy and endocrinology outpatient follow-up were recommended.

The heart and large vessels CT angiography showed postoperative changes of CHD, with an enlarged coronary sinus, residual left superior vena cava, and an enlarged right atrium and right ventricle. There was no obvious patchy shadow in the two lungs. Electrocardiogram examination suggested incomplete right bundle branch block with right ventricular hypertrophy, and ST-segment changes. Ultrasonography of the uterus and ovaries demonstrated that the bilateral adnexal area was slightly thickened, with a thickness of 0.27 cm on the left and 0.23 cm on the right. The gonads were not explored, and the uterus was visible and may have cord-like gonads in thickened bilateral adnexal areas. Ultrasonography of the perineum, pelvic cavity, and groin revealed no testicular echoes. Pituitary MRI suggested a suspected pituitary Rathke fissure while the radiographs of carpal bone showed a bone age of approximately 4.5 years. The ultrasonography of the abdominal and kidneys were normal during our research period, as well as the electroencephalogram.

**Figure 1:**
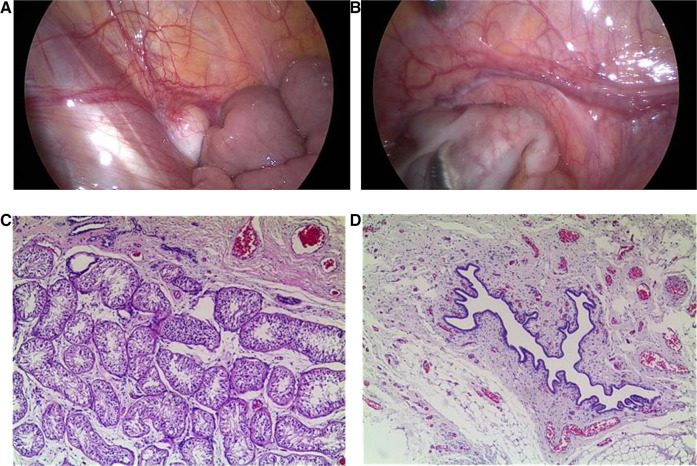
Laparoscopic findings and pathological examination. (**A**) Laparoscopy showed testicular gonads between the lower pole of the right kidney and the opening of the inner ring in the right pelvic cavity. (**B**) Laparoscopy showed muscular tissue in the space between the bladder and rectum in the left pelvic cavity, accompanied by vascular tissue with a slightly enlarged end. (**C**) Pathological examination revealed that the right gonadal tissue was consistent with testicular tissue (×50). (**D**) Pathological examination revealed that the left abdominal mass was consistent with an accessory middle renal duct cyst (×50).

Cystoscopy showed the vagina was sized 4.8 cm, and the cervical opening was visible, no obvious abnormalities in bladder and bilateral ureteral orifices. Laparoscopy showed muscular tissue in the space between the bladder and rectum, from bottom to top, along the left pelvic cavity to the lower pole of the spleen, accompanied by vasculiform tissue with a slightly enlarged end. There was no vas deferens in the right pelvic cavity, a testicular gonad was found between the lower pole of the right kidney and the opening of the inner ring, not well developed, approximately 1.2 × 0.3 cm (Figures 1A,B). That testicular gonad was removed with the consent of the parents. Pathological examination revealed that the left abdominal mass was an accessory middle renal duct cyst, and the right gonadal tissue was testicular (Figures 1C,D). Regular follow-up was done in the urology department.

### Genetic testing

**Figure 2:**
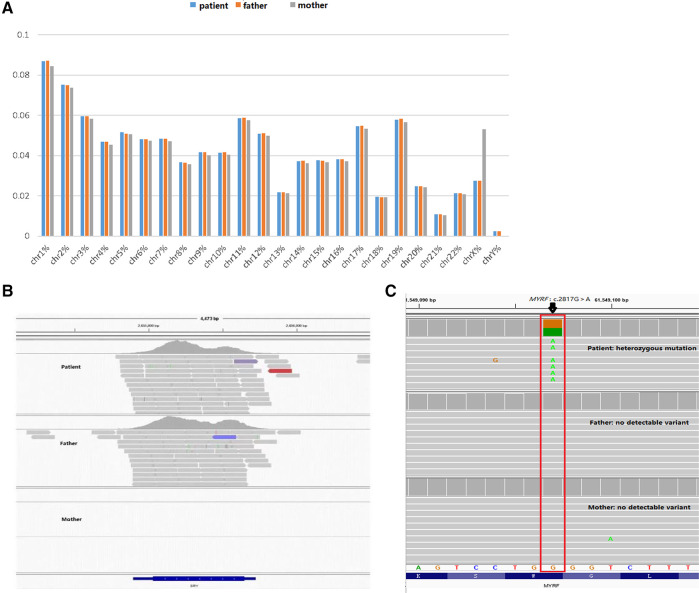
Genetic test results. (**A,B**) Suggests that the patient has a male karyotype (46,XY, carrying SRY). (**C**) c.2817G > A (p.W939*) *de novo* heterozygous mutation in the *MYRF* gene by WES.

**Figure 3:**
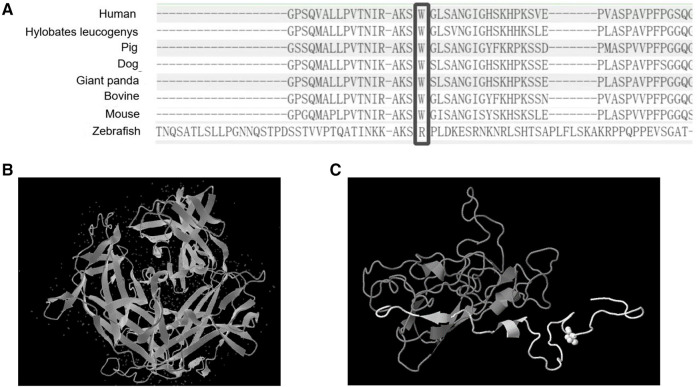
The protein sequence from several organisms and the predicted 3D structure of the variant. (**A**) Comparison of *MYRF* protein sequences from several organisms. (**B**) Normal 3D structure of *MYRF* protein. (**C**) The predicted 3D structure of *MYRF* protein residues 890–1,138 was modeled by Genome3D (residues 890–939 are colored white, and residues 940–1,138 are colored gray). As we could see from the predicted 3D structure, the variant caused a premature termination codon, resulting in protein truncation (approximately 15% of protein length is missing), and damaging the protein structure (region colored in gray).

To further clarify the diagnosis, WES was performed on the DNA of the patient and her unaffected parents after informed consent. DNA was extracted from the peripheral blood of the patient and the parents with normal phenotypes for WES. Copy number variation analysis confirmed the karyotype of 46,XY, carrying SRY (Figures 2A,B). One *de novo* heterozygous variant in *MYRF* was identified, namely c.2817G > A (NM_001127392.3) (Figure 2C), which resulted in early termination (p. W939*). This variation had not been reported or registered in several variant databases, including 1,000 Genomes, GnomAD, LOVD, and HGVD. The ClinVar database annotated it as “pathogenic”, but further literature search revealed no relevant reports. According to the ACMG Classification Standards and Guidelines for Genetic Variations (15), the variant showed strong evidence of pathogenicity because it was a nonsense variant (PVS1), and a *de novo* variant (PS2), and was not included in 1,000 Genomes, GnomAD, LOVD, and HGVD (PM2). Analysis of conserved sequences suggested that this variant was located in highly conserved sequences across several species (PP3) (Figure 3). Finally, we regarded the variant identified in our patient as a pathogenic variant (PVS1 + PS2 + PM2 + PP3) associated with CUGS.

### Literature review

We searched the PubMed database, OMIM, HGMD, and China National Knowledge Infrastructure (CNKI) using “cardiac-urogenital syndrome (CUGS)” “disorders/differences of sex development (DSDs)” and “*MYRF*” as keywords. The search period was from the database's inception to April 30, 2022. Five documents reporting on CUGS and *MYRF* were retrieved (6–10). The clinical characteristics and *MYRF* gene variants of twenty patients (male: 16, female: 4) with CUGS who carried *MYRF* gene variants, as documented in these studies, are summarized in Table 1. The most common clinical presentation of syndromic *MYRF* is as follows: CHD (18/20, 90.0%), genitourinary anomalies (14/17, 82.4%), diaphragmatic anomalies (13/20, 65.0%), and pulmonary hypoplasia (9/19, 47.4%). The rare clinical presentations of CUGS are nanophthalmos (*n* = 1, c.789dupC/p.S264Qfs*74) (10) and short stature (*n* = 1, c.1435C > G/p. L479V) (8).

**Table 1:** The clinical and genetic findings in CUGS cases with *MYRF* variants.

No/Ref	Genotype	Gender	Congenital heart disease	Genitourinary anomalies	Diaphragm anomalies	Pulmonary hypoplasia	Others
1 (6)	c.2336 + 1G > A	M	Scimitar syndrome	Hypospadias, micropenis, cryptorchidism	−	+	Speech delay, alive at 18 months old
2 (6)	c.2518C > T, p. (R840*)	M	Scimitar syndrome	Persistent urachus	CDH	+	Died at 10 days
3 (7)	c.1254_1255dupGA, p. (T419Rfs*14)	M	Hypoplastic left heart syndrome	Ambiguous genitalia	−	+	Intestinal malrotation, pregnancy terminate at 19 weeks
4 (8)	c.235dupG, p. (G81Wfs*45)	M	Tetralogy of Fallot, ASD, VSD	Cryptorchidism	Left CDH	−	
5 (8)	c.1303G > A, p. (G435R)	F	VSD	No internal sex organs, blind-ending vagina	Left CDH	−	Accessory spleen
6 (8)	c.2036T > C, p. (V679A)	M	ASD, VSD	unknown	Left CDH	−	Deceased
7 (8)	c.2084G > A, p. (R695H)	M	Hypoplastic left heart syndrome	Ambiguous genitalia, cryptorchidism	CDH	−	Intellectual disability and motor delay, alive at 2 years old
8 (8)	c.1904-1G > A	F	Scimitar syndrome	unknown	Right CDH	−	Deceased
9 (8)	c.1904-1G > A	F	Hypoplastic left heart syndrome	unknown	−	−	Deceased
10 (8)	c.1786C > T, p. (Q596*)	M	dextrocardia	Female-appearing external genitalia	−	+	−
11 (8)	c.1160T > C, p. (F387S)	M	Aortic arch hypoplasia, coarctation of aorta, hypoplastic left heart syndrome	Ambiguous genitalia, hypospadias, cryptorchidism	−	−	−
12 (8)	c.1209G > C, p. (Q403H)	M	Scimitar syndrome, aortic arch hypoplasia, ASD, bicuspid aortic valve, hypoplastic left heart syndrome, mitral stenosis, VSD	Cryptorchidism	Right hemidiaphragm eventration	+	−
13 (8)	c.1435C > G, p. (L479V)	M	Bicuspid aortic valve, coarctation of aorta	Female-appearing external genitalia	−	−	Short stature
14 (9)	c.3118A > G, p. (R1040G)	M	Scimitar syndrome, hypoplastic left heart syndrome, mitral valve atresia, hypoplastic aortic valve, VSD, ASD, PDA	−	Right diaphragm elevation	+	Alive at 3 months
15 (9)	c.3239dupA, p. (E1081Gfs*5)	M	Hypoplastic left heart syndrome	Hypospadias, cryptorchidism, chordee	Left CDH	+	Died at 4 weeks
16 (9)	c.350_366delinsT, p. (G117Vfs*31)	M	−	Ambiguous genitalia, hypospadias, horseshoe kidney	CDH	−	
17 (10)	c.789dupC, p. (S264Qfs*74)	F	Hypoplastic left heart syndrome		CDH	NA	Died at 3 days
18 (10)	c.789dupC, p. (S264Qfs*74)	M	Hypoplastic left heart syndrome, aortic and mitral valve atresia, aortic arch hypoplasia, coarctation of aorta distal to the left subclavian artery	−	Left CDH	+	Pregnancy terminated
19 (10)	c.789dupC, p. (S264Qfs*74)	M	ASD	Unilateral cryptorchidism, decreased sperm motility	−	−	Nanophthalmos
20 (10)	c.789dupC, p. (S264Qfs*74)	M	−	Ambiguous genitalia	Left CDH	+	Died at 1 day

F, female; M, male; ASD, atrial septal defect; VSD, ventricular septal defect; PDA, patent ductus arteriosus; CDH, congenital heart defects; NA, not available.

Two documents regarding DSDs and *MYRF* were retrieved (11, 16). Seven patients (karyotype 46,XY: 5, karyotype 46,XX: 2) with DSDs carried *MYRF* gene variants; DSD with nanophthalmos (*n* = 1, c.2572 + 1G > A) (16), their clinical characteristics and *MYRF* gene variants are summarized in Table 2.

**Table 2:** Summary of clinical and genetic findings in DSD cases with *MYRF* variants.

No/Ref	Genotype	Karyotype	Social sex	Age	Genital findings	Endocrine findings	Others
1 (11)	c.278del, p. (P93Rfs∗7)	46,XY	F→M at 4 years	14 years	Tanner I; cryptorchidism: abdominal (R) and inguinal (L); hypospadias (perineal type); microphallus: 2.3 cm (−3SD); anterior-positioned and bifid scrotum	At 2 months, baseline T: 125 (120–400) ng/dl, hCG-stimulated T: 440 (> 200) ng/dl	Orchidopexy at 1 year, genitoplasty at 2 years, TE (25 mg i.m.) (age): 3 × (6–10 months), PL increment (cm): 2.3→3.9 (+ 1.1 SD)
2* (11)	c.789del, p. (S264Afs∗8)	46,XX	F	21 years	Tanner III (breast), II (pubis); ovary absent (L), small (R); Fallopian tube absent; uterus absent (L), restiform cervix (R); vagina absent and surrounded by a cyst	At 15 years, baseline LH 45.2 IU/L, baseline FSH 70.1 IU/L, baseline T: 55.0 ng/dl	Genitoplasty at 15 years
3* (11)	c.789del, p. (S264Afs∗8)	46,XX	F	21 year	Tanner II (breast), II (pubis); ovary absent (L), small (R), Fallopian tube: Absent, Uterus: Absent (L); restiform cervix (R), Vagina: Absent, surrounded by a cyst	Age at examination: 15 years, LH (IU/L) baseline: 25.7, FSH (IU/L) baseline: 113.1, T (ng/dl) baseline: 54.9	Genitoplasty at 15 years
4 (11)	c.1642_1666del, p. (A548Tfs∗49)	46,XY	M	4 years 4 months	Tanner I; cryptorchidism; hypospadias (penile type)	At 1 years, baseline LH 0.73 (0.2–1.9) IU/L, FSH 3.91 (*<*0.3–2.4) IU/L; GnRH-stimulated LH 9.49 (1.1–6.0), FSH 21.62 (1.9–7.6) IU/L; baseline T *< *5 (*<*5) ng/dl, hCG-stimulated: 518 (*>* 200); baseline DHT: *<*5 (*<*5) ng/dl, baseline AMH 18.3 (43.3–79.3) ng/ml	Orchidopexy (age): 1 year 7 months, Genitoplasty (age): 2 years 11 months, DHT cream (age): Yes (2 years 8 months), PL increment (cm): Increased (no record), Developmental delay:DQ63, Diaphragmatic hernia (l)
5 (11)	c.1328A *> *C: p. (Q443P)	46,XY	M	2 years 5 months	Tanner stage: Prepubertal, Cryptorchidism: Inguinal (L and R), Hypospadias: Penoscrotal type, Abnormal scrotum: Small and bifid	At 3 m, LH (IU/L) baseline: 8.9 (0.2–1.9), FSH (IU/L) baseline: 8.9 (*<*0.3–2.4), T (ng/dl) baseline: 116 (120–400), DHT (ng/dl) baseline: 20, AMH (ng/ml) baseline: 19.6 (43.3–79.3).$Age at examination: 7 months, LH (IU/L) baseline: 0.3 (0.2–1.9), LH (IU/L) GnRH-stimulated: 8.4 (1.1–6.0), FSH (IU/L) baseline: 3.4 (*<*0.3–2.4), FSH (IU/L) GnRH-stimulated: 23.9 (1.9–7.6), T (ng/dl) baseline: *<*5 (*<*5), T (ng/dl) hCG-stimulated: 78 (*>*200), AMH (ng/ml) baseline: 15.0 (43.3–79.3)	TE (25 mg i.m.) (age): 1 × (5 months), PL increment (cm): 1.5→3.0 (+1.2 SD)
6 (16)	c.313A > G, p. (N105D)	46,XY	F→M at 1 month	birth	Ambiguous genitalia, Perineal hypospadia, Bilateral cryptorchidism:Left testis -inguinal canal; right testis -abdominal cavity.	NA	Testicular biopsy confirmed the presence of testicular tissue with severe stromal sclerosis and tubular atrophy
7 (16)	c.2572 + 1G > A	46,XY	F	14 years	Clitoromegaly, vaginal hypoplasia, One migratory gonad from abdominal cavity to inguinal canal	NA	High-grade hypermetropia 26 years, gonadectomy: lack of germ cells, presence of Sertoli cells and Leydig cell hyperplasia 29 years, vaginoplasty and clitorectomy

2*and 3*: Monozygotic twin; F, female; M, male; LH, luteinizing hormone; FSH, follicle-stimulating hormone; DHT, dihydrotestosterone; TE, testosterone enanthate; PL, penile length; DQ, development quotient measured with Kyoto Scale of Psychological Development; NA, not available.

Previous studies reported a nonsyndromic form of isolated nanophthalmos (*n* = 10, c.789delC/p.S264Afs*8, c.789dupC/p.S264Qfs*74, c.1433G > C/p.R478P, c.2956C > T/p.R986X, c.3377delG/p.G1126Vfs*31, c.3194 + 2T > C, and c.3274_3275delAG/p.L1093Pfs*22) (10, 13, 17), nanophthalmos with mitral valve prolapse, unilateral cryptorchidism and micropenis (*n* = 1, c.789dupC/p.S264Qfs*74) (12), nanophthalmos with unilateral cryptorchidism and ASD (*n* = 1, c.789dupC/p.S264Qfs*74) (10), nanophthalmos with posterior fossa cyst (*n* = 1, c.1553C > T/p.T518M) (18), and 2 large nanophthalmos families with or without dextrocardia or CDH (c.789dupC/p.S264Qfs*74, c.3376-1G > A, and c.3361delC/p.R1121Gfs*36) (12, 19), their clinical characteristics and *MYRF* gene variants are summarized in Table 3.

**Table 3:** Summary of clinical and genetic findings in nanophthalmos cases with *MYRF* variants.

No/Ref	Genotype	Gender	Age	Nanophthalmos	Congenital heart disease	Genitourinary anomalies	Diaphragm anomalies	Others
1 (10)	c.789dupC, p. (S264Qfs*74)	F	newborn	+	−	−	−	
2 (10)	c.789dupC, p. (S264Qfs*74)	M	33 years	+	ASD	Unilateral cryptorchidism and decreased sperm motility	−	
3 (12)	c.789dupC, p. (S264Qfs*74)	M	8 years	+	Mitral valve prolapse	Unilateral cryptorchidism and micropenis	−	
4* (12)	c.3376-1G > A			+	4 individuals with dextrocardia	−	−	
5 (13)	c.3377delG, p. (G1126Vfs*31)	M	29 years	+	−	−	−	Angle-closure glaucoma in right eye at 38 years old
6 (13)	c.3377delG, p. (G1126Vfs*31)	F	6 years	+	−	−	−	
7 (13)	c.3377delG, p. (G1126Vfs*31)	F	NA	+	−	−	−	
8 (13)	c.3274_3275delAGp. (L1093Pfs*22)	M	6 years	+	−	−	−	
9 (13)	c.3194 + 2T > C	F	40 years	+	−	−	−	
10 (17)	c.789delC, p. (S264Afs∗8)	F	16 years	+	−	−	−	Poor eyesight from an early age, amblyopia at the age of 7 years.
11 (17)	c.789dupC, p. (S264Qfs*74)	F	30 years	+	−	−	−	Blurred eyes for more than 20 years and a rapid decline in vision of right eye at 30-years old.
12 (17)	c.1433G > C, p. (R478P)	F	35 years	+	−	−	−	
13 (17)	c.2956C > T, p. (R986X)	M	32 years	+	−	−	−	
14 (18)	c.1553C > T, p. (T518M)	M	3 years	+	−	−	−	Posterior fossa cyst
15* (19)	c.3361delC, p. (R1121Gfs*36)			+	2 individuals with dextrocardia	−	One with right CDH	

F, female; M, male; ASD, atrial septal defect; CDH, congenital heart defects; NA, not available. 4*and 15*: two large nanophthalmos families.

## Discussion

**Figure 4:**
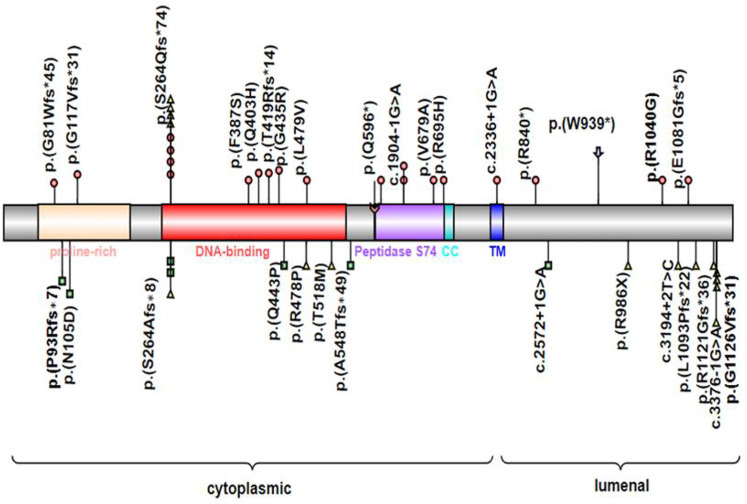
*MYRF* protein schematic, showing the location of individual domains, and relative positions of reported disease-associated variants associated with CUGS (colored circles) or DSDs (colored squares) or nanophthalmos or high hyperopia (colored triangles). Symbol (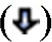) indicates the variant in this study. CC, coiled-coil domain; TM, transmembrane domain; Symbol (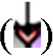) indicates the autolytic cleavage site.

*MYRF* consists of the N-terminal proline-rich domain, DNA binding domain (DBD), intramolecular chaperone autoprocessing (ICA), or peptidase S74 domain in the cytoplasm, transmembrane helix, and luminal domain in the lumen of the endoplasmic reticulum. In these conserved domains, the DBD directly binds to the promoters of target genes, and the ICA domain triggers the homotrimerization of *MYRF* protein and subsequently results in autoproteolytic cleavage to release an N-terminal transcriptional activator translocating to the nucleus, which is important for transcriptional activation (20, 21). Mutations could be present in any domain of *MYRF* and result in haploinsufficiency or single amino acid changes, and single amino acid changes were mostly located in the DNA binding domain and peptidase S74 domain. Both syndromic presentations and nanophthalmos or high hyperopia were associated with missense, nonsense, frameshift, and essential splice variants in *MYRF* (6–9, 11, 19) (Figure 4).

Here, we reported the case of a 4-year-7-month-old “girl” who presented with multiple clinical manifestations, including VSD, ASD, PDA, persistent superior vena cava, pulmonary hypertension, female external genitalia with a slightly enlarged clitoris, uterus and vagina with no ovary, 46,XY karyotype, vanishing testis with testicular gonadal tissue in the right pelvis, vasculiform tissue in the left pelvis, Tanner stage I (breast) and I (pubis), short stature, and amblyopia. Syndromic symptoms of CUGS and 46,XY DSD were overlapping. Exome sequencing revealed a heterozygous *de novo* c.2817G > A (p.W939*) nonsense mutation of *MYRF* in exon 22. Analysis of conserved sequences suggested that this variant was located in highly conserved sequences across several species. A DECIPHER database search revealed that the variant caused haploinsufficiency (%HI = 40.26) and loss-of-function (pLI = 1.00). The mutation generated a premature termination codon, leading to protein truncation (approximately 15% of the protein length was missing) and damage to the protein structure (22) (Figure 3).

Previously, 30 pathogenic variants of *MYRF* had been described in patients with CUGS, DSDs, and nanophthalmos. The syndromic CUGS and DSDs *MYRF* variants were *de novo*, whereas variants with nanophthalmos or high hyperopia showed kindred transmission. Twenty cases of CUGS with structural congenital disabilities affecting the heart, lungs, diaphragm, and genitourinary system had been reported to carry variants of *MYRF*, including frameshift (*n* = 8), missense (*n* = 7), splice site (*n* = 3), and nonsense (*n* = 2) variants. Seven patients with DSDs showed *MYRF* variants, including 4 frameshifts, 2 missense mutations, and 1 splice site. Most of these variants (17/22) affected the 5′ end of the transmembrane domain (TM) in *MYRF*, except the variant p.(E1081Gfs*5) closest to the *C*-terminal reported in a syndromic case, which lies in exon 25. This suggested that loss of *MYRF* function, triggering nonsense-mediated mRNA decay was the likely mode of action of these syndromic variants. We also observed male predominance in the published cases of CUGS (16/20) and DSDs (5/7), with genitourinary anomalies present in most of the cases. This also suggested that loss of function of the *MYRF* transcription factor leads to the transcriptional dysregulation of genes related to sex development (11). Further studies are warranted to confirm this hypothesis. Compared with the variants presenting severe syndromic forms, the variants that manifested nanophthalmos occurred in the final six exons of *MYRF* or their associated splice sites (after exon 22), except for the variants c.789delC (p.S264Afs*8) and c.789dupC (p.S264Qfs*74). The c.789dupC (p.S264Qfs*74) had been observed in variable phenotypic manifestations (10, 12, 17), while c.789delC (p.S264Afs*8) had only been described in the milder clinical presentation of DSDs and isolated nanophthalmos in previous reports (11, 17), although they had the same position of *de novo* mutations. Therefore, we agree that severe syndromic manifestations are relevant to *MYRF* haploinsufficiency, while isolated nanophthalmos may sometimes affect *MYRF* homotrimerization, autoproteolysis, or transcriptional activity (19).

Previous study also considered that introduction of a premature termination codon in the terminal exon may cause the variant at the 3′ end of the penultimate exon of *MYRF* avoiding nonsense-mediated decay (23). Therefore, the isolated nanophthalmos and incompletely penetrant dextrocardia (12) or high hyperopia were possibly associated with further downstream variants. However, in our report, the variant at the 3′ end of exon 22 caused a genetic syndrome of CUGS and DSD, similar to the variants c.3118A > G (p.R1040G) in exon 23 and c.3239dupA (p. E1081Gfs*5) in exon 25 that Rossetti et al. (9) reported, which also are likely to trigger nonsense-mediated mRNA decay. This suggests that loss of *MYRF* function, even in the last C-terminal exons, is still the most likely mode of action of these variants. Besides, Garnai et al. (12) considered *MYRF* as a dosage-sensitive transcription factor, because the ocular tissues were more sensitive than the cardiac and urogenital tissues, especially to changes in the *C*-terminus of the protein. In our case amblyopia was also observed. Since strabismus and amblyopia in early childhood may develop to uncorrected high hyperopia (17), further follow-up is required for subsequent visual loss. Further, it is possible that ocular symptoms have not yet been observed in individuals with *MYRF* variants causing severe syndromic symptoms, because they did not survive infancy and owing to plausible lack of detailed ocular examination. Despite these assumptions, the detailed action mode of the C-terminal region in *MYRF* remains unknown. Deeper research is necessary to determine the mechanisms that explain these individuals' ocular and systemic phenotypes.

In summary, we reported a 4-year-7-month-old patient presenting multiple clinical manifestations with syndromic symptoms of CUGS and 46,XY DSD, ocular symptoms, and short stature. WES identified a novel truncation mutation in the 3′ end of *MYRF*, located in exon 22. Our findings suggest further studies to illustrate the function of the critical *MYRF* as well as how different variants affect the function of *MYRF* in different cell types in human disorders are necessary.

## Data Availability

The original contributions presented in the study are included in the article/Supplementary Material, further inquiries can be directed to the corresponding author/s.
